# In vitro and in vivo fermentation models to study the function of dietary fiber in pig nutrition

**DOI:** 10.1007/s00253-024-13148-9

**Published:** 2024-04-29

**Authors:** Weikang Huangfu, Shixi Cao, Shouren Li, Shuhang Zhang, Mengqi Liu, Boshuai Liu, Xiaoyan Zhu, Yalei Cui, Zhichang Wang, Jiangchao Zhao, Yinghua Shi

**Affiliations:** 1 College of Animal Science and Technology, Henan Agricultural University, Zhengzhou, No.15 Longzihu University Area, Zhengdong New District, Zhengzhou, 450046 China; 2 Henan Key Laboratory of Innovation and Utilization of Grassland Resources, Zhengzhou, China; 3 Henan Forage Engineering Technology Research Center, Zhengzhou, 450002 Henan China; 4 Department of Animal Science, Division of Agriculture, University of Arkansas, Fayetteville, AR USA

**Keywords:** Dietary fiber, Gut microbes, SCFAs, Intestinal gas, In vitro fermentation, In vivo fermentation

## Abstract

**Abstract:**

The importance of dietary fiber (DF) in animal diets is increasing with the advancement of nutritional research. DF is fermented by gut microbiota to produce metabolites, which are important in improving intestinal health. This review is a systematic review of DF in pig nutrition using in vitro and in vivo models. The fermentation characteristics of DF and the metabolic mechanisms of its metabolites were summarized in an in vitro model, and it was pointed out that SCFAs and gases are the important metabolites connecting DF, gut microbiota, and intestinal health, and they play a key role in intestinal health. At the same time, some information about host-microbe interactions could have been improved through traditional animal in vivo models, and the most direct feedback on nutrients was generated, confirming the beneficial effects of DF on sow reproductive performance, piglet intestinal health, and growing pork quality. Finally, the advantages and disadvantages of different fermentation models were compared. In future studies, it is necessary to flexibly combine in vivo and in vitro fermentation models to profoundly investigate the mechanism of DF on the organism in order to promote the development of precision nutrition tools and to provide a scientific basis for the in-depth and rational utilization of DF in animal husbandry.

**Key points:**

• *The fermentation characteristics of dietary fiber in vitro models were reviewed.*

*• Metabolic pathways of metabolites and their roles in the intestine were reviewed.*

*• The role of dietary fiber in pigs at different stages was reviewed.*

**Graphical Abstract:**

**Figure:**
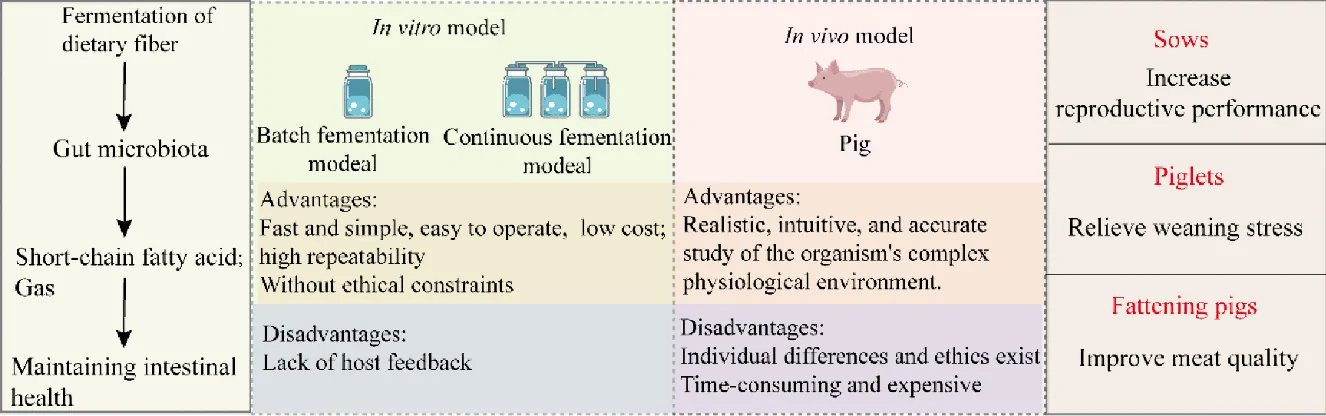

## Introduction

Dietary fiber (DF) is a general term for polysaccharides, oligosaccharides, and non-polysaccharides in plant foods that are not digested or absorbed by the animal body and is classified as soluble dietary fiber (SDF) and insoluble dietary fiber (IDF) based on its solubility and fermentation properties (Gill et al. 2021). SDF can dissolve in water to form a viscous gel, not digested and absorbed by the small intestine. After entering the large intestine, it is easy to be rapidly fermented and degraded by microbes into metabolites, mainly including β-glucan, arabinoxylan, and pectin, and the feed sources are such as inulin and beetroot residue (Makki et al. 2018). IDF includes lignin, cellulose, and resistant starch, and the primary sources are bran, alfalfa, oat hulls, etc., which have a limited ability to ferment and enhance the health of intestines by promoting intestinal peristalsis (McRorie et al. 2017). DF-rich feeds have lower nutritional value than high-starch or protein diets due to lower digestible energy or amino acid levels (Woyengo et al. 2014). Therefore, DF is rarely added to monogastric animal feeds. However, with nutritional studies, it has been found that DF-enriched feeds prevent intestinal diseases (constipation, diarrhea, etc.) and play a key role in maintaining normal physiological functions and promoting digestion and metabolism by improving the animal’s intestinal microbes and their metabolites (Chen et al. 2018). Therefore, understanding the physiological functions of different DFs in pig nutrition and the composition of their metabolites can help in precision feeding.

The gut microbiota is the most complex micro-ecosystem in the animal body, which plays a crucial role in regulating animal health by interacting with the nutrients in the diet (Makki et al. 2018). These include promoting nutrient absorption, participating in metabolism, maintaining the intestinal barrier, enhancing the immune system, and enhancing resistance to pathogens (Gilbert et al. 2016). At the same time, the gut microbiota is also regulated by changes in the physiological state of the host, including genetics, host physiology, dietary, and various environmental factors (Blander et al. 2017). As a novel diet composition, DF positively affects intestinal health by modulating the gut microbiota composition and metabolites produced by fermentation, such as short-chain fatty acids (SCFAs) and gases (Levy et al. 2017). Therefore, understanding the effects of different DFs on the gut microbiota and metabolites, as well as the metabolic mechanisms of the metabolites, is crucial for maintaining the health of the animal organism.

The relationship between DFs and animal health and the modulatory effects of various DFs on the intestinal microbiota have attracted much attention (Holscher 2017). In vitro and in vivo models are commonly used to study this relationship (Ye et al. 2022a). Kang et al. (2022) reviewed the progress of DF in vitro and in vivo fermentation modeling, as well as the relationship between DF and the gut microbiota. Li et al. (2021) described the physiological functions of DF in pig nutrition and its application prospects. However, the fermentation products of fiber and their physiological functions and regulatory mechanisms were not mentioned. Ma et al. (2022b) further elaborated on the effects of gut microbes on the host and the mechanism of action of SCFAs on the barrier function of the organism but did not mention the issue of gas production and function. Therefore, a comprehensive understanding of the metabolic patterns and mechanisms of action of different types of DF on the gut microbiota and their metabolites is essential for maintaining the health of the animal. This paper aims to review the fermentation characteristics of different DF components through in vitro simulation, understand the metabolic processes and regulatory mechanisms of metabolites, and focus on the effects of SCFAs and gases on host health. The roles played by DF and its metabolites in different stages of pigs were further reviewed. Finally, the advantages and disadvantages of different models were compared so that the combined in vitro and in vivo fermentation models could be applied appropriately in future studies to investigate in depth the mechanism of action of different DFs for more precise feeding strategies, which will help to promote the development of the field of animal nutrition.

## In vitro fermentation modeling to study the effects of DF on pigs

With the increasing number of digestive disorders caused by improper diet, there has been a growing interest in the effects of nutrients in food on animal health. However, the effect of nutrients on the organism’s health depends not only on the digestive process of the nutrients in the animal’s digestive tract but also on the function of the nutrients through the production of various small molecules (Song et al. 2019). The in vitro model has the unique advantage of mimicking the composition and activity of microbes in the gastrointestinal tract, is relatively simple in structure, has no ethical constraints, and can be effectively controlled to avoid interference from other components. In addition, it allows for dynamic sampling in time and has therefore been proposed as an alternative to in vivo studies (Jeong et al. 2019). In recent years, many microbiologists and nutritionists have developed various in vitro intestinal fermentation models for simulating the digestion of food or its single components in animals (Nissen et al. 2020). Therefore, we reviewed the fermentation characteristics, metabolic pathways of metabolites, and mechanisms of action of different DFs, which are essential for studying the effects of DFs on organismal health.

## In vitro modeling to study the fermentation properties of DF

In vitro gut modeling provides a cost-effective way to study the interactions between DF and the gut microbiota. Such models can rigorously simulate and control intestinal physiological parameters such as pH, temperature, residence time, medium composition, and anaerobic bacteria. At the same time, they can record metabolite changes in real-time (Cheng et al. 2022). Various in vitro digestion models have been developed, mainly classified into static and dynamic models. Static models mainly include oral, gastric, small intestinal, and colonic stages, and most static models complete several stages of digestion in a specific reaction vessel, with pH, enzymes, and electrolytes fixed for each stage (Kang et al. 2022). Researchers have developed in vitro dynamic models to compensate for the shortcomings of static models. The in vitro dynamic fermentation model usually consists of a fermenter, thermostat controller, stirrer, gas exchange device, etc. It can simulate the gastrointestinal environment under different conditions and use a computer to control the digestive fluid, enzymes, pH value, duration, peristalsis, absorption and emptying parameters during digestion to be closer to the physiological environment in vivo (Ji et al. 2022; Moon et al. 2016). However, most DFs are indigestible under simulated salivary, gastric, and small intestinal conditions and maintain relatively good biological activity (Shao et al. 2022). In vitro static batch fermentation models are commonly used to examine the metabolism of different DFs by individual bacterial strains or mixed cultures of animal gut microbiota in sealed test tubes or reactors under confined anaerobic environments (Bohn et al. 2018). Thus, they effectively predict the in vivo digestive outcome of DFs. On this basis, it can be used to study the fermentation rates of different DF components and their metabolites, such as cellulose, arabinoxylan, xyloglucan, and β-glucan (Jonathan et al. 2012; Williams et al. 2011). In addition, the effects of other DF sources, prebiotics, and other food components on microbial, SCFAs, and gas production characteristics have also been investigated (Loo et al. 2022; Xie et al. 2022; Zhang et al. 2023). In conclusion, in vitro static batch fermentation models are widely used for their simplicity and rapidity in studying the interaction between DF and gut microbes.

Many studies have been carried out on the fermentation characteristics of DF, changes in metabolites, and patterns of interactions between microbes using in vitro batch fermentation techniques. These studies have focused on fiber fractions such as lignin, cellulose, pectin, β-glucan, and xyloglucan, as well as on traditional fiber feedstocks such as wheat bran, oat bran, and sugar beet meal (Tao et al. 2019). Most of these traditional DFs consist of hybrid glycans with different fiber components, which can lead to significant differences in fermentation properties, ability to regulate intestinal microecology and health due to differences in the complex structure and composition of the functional units of the DFs, as well as the diversity of molecular structures such as glycosidic bonding patterns and molar mass (Jonathan et al. 2012). Fiber components, as the basic structural and functional units of DF, have more stable fermentation properties and functional roles and are directly linked to the functional regulation of DF. Table 1 summarizes the fermentation characteristics of fiber components and provides a scientific basis for the rational use of DF in animal organisms. Among the fiber fractions, polysaccharides such as arabinoxylan, pectin, and β-glucan belong to the fast-fermenting fibers (Bai et al. 2021; Williams et al. 2011). Cellulose and glucomannan belong to the slow-fermenting fiber fractions (Mikkelsen et al. 2011). Similarly, there were significant differences in the fermentation characteristics of traditional fibers. Bai et al. (2020) investigated the fermentation characteristics of growing pigs, in which oat bran showed the highest fermentation rate and produced more SCFAs and gas, which was related to the high content of starch and SDF in oat bran. The slow fermentation of soybean hulls was associated with a high fiber content. The fermentation characteristics of konjac flour (containing mainly konjac glucan) and lignocellulose (containing mainly cellulose) were investigated using sow fecal microorganisms and were challenging due to the high cellulose content of the lignocellulose; the high fermentation rate of konjac flour was associated with its high content of SDF (Pi et al. 2021). In summary, fibers enriched with high SDF are more beneficial for fermentation than fibers enriched with IDF fractions.

**Table 1:** Fermentation characteristics of different fiber groups studied using in vitro batch fermentation models

Growth phase	DF	Fermentation rate	Mechanism			References
			SCFAs	Gas	Gut microbiota	
Sows	β-glucan	Fast	Acetate↑ Butyrate↑	↑	N/A	(Jonathan et al. 2012; Williams et al. 2011)
	Glucomannan	Slow	Acetate↑ Propionate↑	↑	N/A	(Jonathan et al. 2012)
	Cellulose	Slow	Propionate↑	↑	N/A	(Jonathan et al. 2012)
	Pectin	Slow	Acetate↑ Propionate↑	↑	N/A	(Jonathan et al. 2012)
Growing pigs	Arabinoxylan	Fast	Acetate↑ Propionate↑	↑	N/A	(Jonathan et al. 2012; Mikkelsen et al. 2011; Williams et al. 2011)
	Cellulose	Slow	Acetate↑ Propionate↑	↑	N/A	(Jonathan et al. 2012; Mikkelsen et al. 2011)
Growing pigs	Cellulose	Slow	Propionate↑ Butyrate↑	↑	*Prevotellaceae_NK3B31_group*↑ *Lachnospiraceae XPB_1014_group*↑ *Prevotella_1*↑	(Bai et al. 2021)
	Arabinoxylan	Fast	Propionate↑ Butyrate↑	↑	*Prevotella_9*↑ *Lachnospiraceae XPB_1014_group*↑ *Prevotella_1*↑ *Bacteroides*↑ *Anaerovibrio*↓	(Bai et al. 2021)
	β-glucan	fast	Acetate↑ Propionate↑ Butyrate↑	↑	*Prevotella_9*↑ *Bacteroides*↑ *Anaerovibrio*↓	(Bai et al. 2021)
	Glucomannan	Slow	Acetate↑ Propionate↑ Butyrate↑	↑	*Prevotella_9*↑ *Prevotellaceae_NK3B31_grou*p↑ *Prevotella_1*↑ *Anaerovibrio*↓	(Bai et al. 2021)
Growing pigs	Oat bran	Fast	Acetate↑ Propionate↑ Butyrate↑	↑	*Enterococcus*↑	(Bai et al. 2020)
Growing pigs	Soybean hulls	Slow	Acetate↑ Propionate↑ Butyrate↑	↑	N/A	(Bai et al. 2020)
Sows	Konjac flour	Fast	Propionate↑ Butyrate↑	↑	*Anaerovibrio*↑ *Proteiniclasticum*↑	(Pi et al. 2021)
	Lignocellulose	Slow	Butyrate↑	↑	*Fibrobacter*↑	(Pi et al. 2021)

*N/A* not available

DF is a hybrid glycan composed of different fiber components. Differences in fiber composition, monosaccharides, and glycosidic bonding can lead to significant differences in the fermentation characteristics, intestinal microecological regulation, and health promotion of DF. Therefore, DF’s composition and properties should be considered when formulating rational feeds to maximize the intestinal health of pigs.

## In vitro fermentation modeling to study metabolite synthesis and distribution

**Fig. 1:**
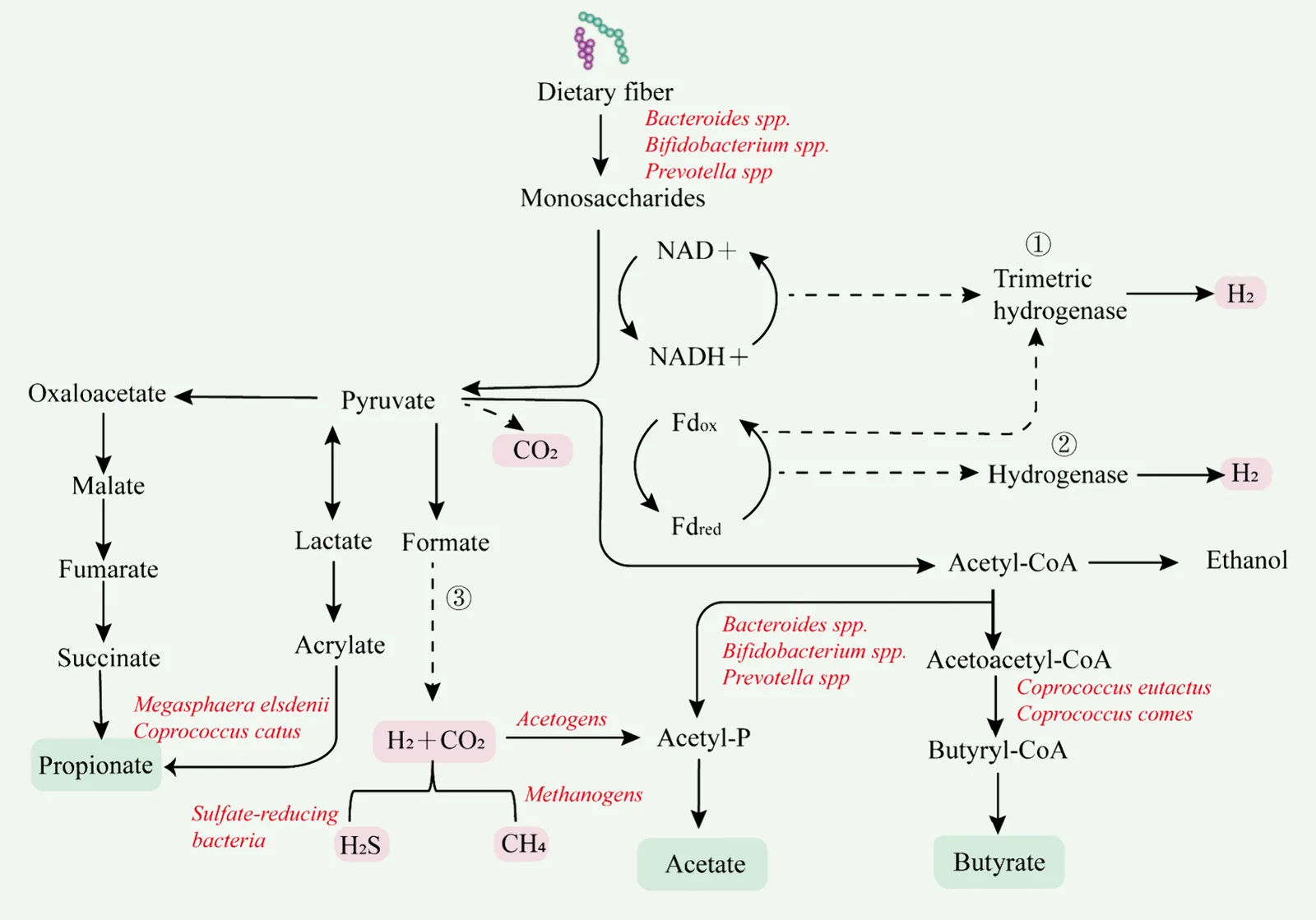
DF produced gas, and SCFAs metabolic pathways by microbial fermentation, and a red box represented the gas; the green box represents SCFAs. H_2_ is mainly produced in three ways. ① Generated by oxidation of reduced flavin (FADH) and nicotinamide adenine dinucleotides (NADH) by microbial hydrogenases. ② Pyruvate-erredoxin oxidoreductase and hydrogenase produce hydrogen from pyruvate. ③ Formate produces H_2_ and CO_2_ under the action of formate hydrogenase. Fdox, oxidized flavin adenine dinucleotide; Fdred, reduced flavin adenine dinucleotide. CO_2_ can be produced by the cleavage of pyruvate to formate or by the metabolism of formate hydrogenase. CO_2_ and H_2_ are produced by CO_2_ and H_2_ metabolism. The production pathway of SCFAs (acetate, propionate, and butyrate). The production pathway of SCFAs (acetate, propionate, and butyrate). Acetate is produced by the fermentation of various microbes, such as *Bacteroides*, *Bifidobacterium*, and *Prevotella*, via pyruvate through the Acetyl-CoA pathway. Propionate is the primary metabolite of *Bacteroides* fermentation, mainly produced through the succinic acid and acrylate pathways. Butyrate, a metabolite of *Firmicutes*, is reduced from Acetyl-CoA to Butyryl-CoA, which is converted to butyrate by transbutyrylase and butyrate kinase. The dotted line indicates that the pathway is still a theoretical hypothesis. The dotted line indicates that the pathway is still a theoretical hypothesis (Comino et al. 2018; Maccaferri et al. 2010; Wang et al. 2019a)

In recent years, with the rapid development of sequencing technology, it has been gradually recognized that gut microbes and their metabolites are closely associated with various diseases (Zmora et al. 2019). Figure 1 describes in detail the process in which DF is degraded into monosaccharides by microbial degrading enzymes and then metabolized into SCFAs (acetate, propionate, butyrate) and gases (H_2_, CO_2_, CH_4_, H_2_S) through glycolysis or pentose phosphate pathway (LeBlanc et al. 2017). SCFAs positively influence the maintenance of gut microbial homeostasis and promote intestinal health (Gill et al. 2018). In recent years, gas has also been gradually explored and plays a vital role in intestinal health (Kalantar-Zadeh et al. 2019). Due to uncontrollable factors such as the rapid uptake of fermentation products in vivo, in vitro fermentation modeling to qualitatively and quantitatively determine metabolite synthesis and distribution is a good strategy for the future.

## Role of metabolites in the intestinal tract

### Impact of gas on gut health

Gas is one of the essential products of microbial metabolism and reflects the activity of intestinal microbes and the body’s health status to a certain extent (Pimentel et al. 2013). For example, intestinal diseases such as indigestion and lactose intolerance can be diagnosed by detecting intestinal gas (Gasbarrini et al. 2009). A small part of the gas can enter the capillaries through the epithelial cells of the large intestine or play a specific role in the intestine. In contrast, most of the gas and undigested nutrients are excreted through the anus (Mutuyemungu et al. 2023). Thus, intestinal gas is a microbial fermentation product and an essential medium for animal physiological activities. This paper reviews the effects of intestinal gas on monogastric animals’ health from the aspects of metabolic process, biological activity, and nutritional regulation. This will help us better understand intestinal microbes’ interaction with body health and provide a scientific basis for improving animal health and production efficiency.

#### Hydrogen

H_2_ is the main gas produced by colonic fermentation, which is only produced by microbial fermentation of indigestible substrates (Naito et al. 2018). The most abundant bacteria producing H_2_ include *Bacteroides*, *Ruminococcus*, and *Roseburia* (Mutuyemungu et al. 2023). In addition, excessive H_2_ concentration can hinder the fermentation of bacteria. Therefore, about one-third of H_2_ in the intestine will be metabolized by different hydrogen-nourishing bacteria. These hydrogen-utilizing microbes mainly include methanogens, sulfate-reducing bacteria, and acetate-producing bacteria, which produce CH_4_, H_2_S, and acetate (Nakamura et al. 2010). These play their respective roles in the intestinal ecosystem and help maintain a stable and healthy intestinal environment.

H_2_ plays an important role in improving gut health. It has been shown to have potent anti-inflammatory, antioxidant, and anti-apoptotic activities in various disease models and repair gut barrier function (Bai et al. 2016). H_2_ has been reported to reduce oxidative stress and inflammation-induced injury by modulating the nuclear factor kappa-B (NF-κB) and nuclear factor erythroid 2-related factor 2 signaling pathway (Kura et al. 2018; Yu et al. 2019; Yuan et al. 2018). In addition, many animal models have also verified these effects of H_2_ by drinking hydrogen-rich water. Hydrogen-rich water slows the intestinal barrier function in a mouse model of inflammatory bowel disease induced by sodium dextran sulfate (Ge et al. 2022; Song et al. 2022). Also, the consumption of hydrogen-enriched water increased the SCFAs-producing microbes (*Lachnospiraceae*, *Rikenellaceae*, and *Prevotella*) in the intestine. It reorganized colonic cell metabolism through the H_2_-intestinal microbiota-SCFAs axis to improve intestinal barrier function (Ge et al. 2022). In addition, the latest reports have proved that H_2_ can promote butyrate production (Campbell et al. 2023). Similarly, in the Fusarium toxin-induced intestinal injury model of piglets, oral hydrogen-rich water can improve the apoptosis of intestinal epithelial cells and maintain the intestinal barrier (Ji et al. 2019). It was verified in cell experiments (Ji et al. 2020). In addition, dietary regulation can also promote the production of H_2_ in the intestine. In vitro models have found that fibers such as inulin, pectin, and oligofructose have the effect of fermenting to produce H_2_ (Nishimura et al. 2013; Yu et al. 2020b). Therefore, H_2_ is essential in resisting inflammation and oxidative stress to improve the intestinal barrier. Further research on microbial hydrogen metabolism is helpful in preventing, diagnosing, and managing various intestinal diseases and provides new strategies.

#### Carbon dioxide

CO_2_ is one of the leading gases produced by microbial fermentation of DF in the distal small intestine and colon, and the microbes mainly involved in CO_2_ production are *Firmicutes* and *Bacteroidetes* (Mutuyemungu et al. 2023). This CO_2_ is passively absorbed into the circulation through the colonic mucosa and rapidly eliminated through respiration. In addition, unabsorbed CO_2_ produces CH_4_ by farting or by intestinal microbial metabolism. CO_2_ is an inert gas. A large amount of CO_2_ can produce mechanical stimulation on the intestinal wall through the volume effect, and no other apparent biological effects have been found (Modak 2009). This suggests that CO_2_ may influence the maintenance of intestinal health, although further studies are needed to clarify this aspect of its role.

#### Methane

CH_4_ is produced by methanogens (such as archaea) in the intestine by metabolizing CO_2_ and H_2_ (Triantafyllou et al. 2014). These methanogenic bacteria mainly exist in the colon of animals. Because it can use the final products of bacterial fermentation (such as H_2_, CO_2_, formate, ethanol), it is of great significance to reduce the hydrogen partial pressure in the intestine, maintain the efficiency of microbial fermentation, and maintain the normal physiological function of the intestine (Luo et al. 2017).

CH_4_ in the animal gut is mainly produced by methanogens, which are anaerobic bacteria. However, the critical question of whether methanogens are pathogenic or beneficial remains to be answered (Mutuyemungu et al. 2023). As a hydrogenotrophic microorganism, methanogens can fully use H_2_ in the intestine, promote more thorough fermentation of hindgut bacteria, and produce more SCFAs to participate in host energy metabolism (Million et al. 2016). It has also been reported to have antioxidant and anti-apoptotic effects in animal models of ischemia-reperfusion injury (Li et al. 2017). However, methanogenic bacteria are strongly associated with gastrointestinal diseases (e.g., inflammatory bowel disease, colorectal cancer) (Conway and Macario 2009). Patients with inflammatory bowel disease have relatively low numbers of methanogens in the gut and relatively low concentrations of exhaled methane, possibly due to a reduction in the total number of gut microbes due to diarrhea (Chaudhary et al. 2018). In contrast, methane bacteria were more abundant in the gut of severely constipated anorexia nervosa patients. It was demonstrated that high concentrations of CH_4_ inhibit gastrointestinal motility and cause constipation (Chatterjee et al. 2007). In addition, the presence of CH_4_ reduces the volume of its “parent” molecules (H_2_ and CO_2_) by approximately 20%, thereby reducing the volume effect and inhibiting gastrointestinal peristalsis, which is strongly associated with slow transit constipation (Chatterjee et al. 2007). In conclusion, the effects of methane produced in the intestines on the human body are multifaceted, and the relationship between methanogens and the organism’s health should be studied in the future.

#### Hydrogen sulfide

H_2_S is mainly produced by sulfate-reducing bacteria, including *Desulfotomac-ulum*, *Desulfobulbus*, *Desulfomicrobium*, *Desulfobacter*, and *Desulfomonas* using sulfate as the terminal electron acceptor and H_2_ as the terminal electron donor (Kalantar-Zadeh et al. 2019). In addition, fiber fermentation produces less H_2_S, mainly from the production of sulfur-containing amino acids in proteins. High concentrations of H_2_S have toxic effects on human tissues, especially in the presence of NO, and may disrupt β-oxidation, lipid, and protein synthesis (Chatterjee et al. 2007; Roediger 2008). Studies have also found that H_2_S in the intestine is closely related to intestinal inflammatory diseases and colorectal cancer (Huycke and Gaskins 2004; Ye et al. 2022b). However, when the concentration of H_2_S is low, it also has essential cell signal transduction characteristics (Tomasova et al. 2016). As a gas transmitter, it may help regulate intestinal cellular processes such as inflammation, motility, epithelial secretion, and nociception (Cirino et al. 2023; Dilek et al. 2020). Therefore, future studies need to explore further how the cell signaling properties of H_2_S can be exploited to improve gut health and prevent intestinal inflammatory diseases and colorectal cancer.

Currently, there are still some limitations in the detection methods of intestinal gas. The standard method is to indirectly reflect the gas metabolism by measuring the gas discharged from respiration and the anus. However, this method cannot accurately reflect the changes of gas in the intestine in real-time. Another method is to collect gas by inserting a hose into the anus. This method is relatively accurate but invasive and can cause discomfort (Freire et al. 2022). With the update of gas detection methods, a more accurate and safe gas measurement method, a gas sensing capsule, has emerged in recent years. The capsule can monitor the gas changes in the digestive tract in real-time by animal swallowing. However, the cost of this method is high, and its operation and the data processing process still need to be standardized (Kalantar-Zadeh et al. 2018). In conclusion, research on the mechanisms of production and regulation of intestinal gases in animals and their effects on intestinal diseases and health is still in its infancy. However, it is increasingly recognized that dietary structure can modify the structure of intestinal gases by altering the microbial composition. Therefore, it is important to regulate the relationship between diet composition and intestinal gases by nutritional means or to form individualized feed formulations based on gas composition. It will help prevent and treat gastrointestinal diseases in animals and is a new area of research in animal nutrition.

### Impact of SCFAs on intestinal health

SCFAs are involved in the body’s energy metabolism and supply energy to intestinal epithelial cells (Morrison and Preston 2016; van der Hee and Wells 2021). They also act as signaling molecules to regulate the function of the intestinal mucosal barrier to improve intestinal health (Chen et al. 2020b). After being transported to cells, SCFAs can regulate gene expression through histone deacetylases (HDACs) and play an immunomodulatory role, or SCFAs bind to G-protein coupled receptors, GPRs (e.g., GPR43, GPR41, and GPR109A) receptors on the corresponding cells, sending out signals that activate signaling cascade reactions controlling immune function to play various modulatory roles in the gut (van der Hee and Wells 2021). As the body’s first barrier against invasion by foreign pathogens, the intestinal barrier includes biological, chemical, mechanical, and immune barriers, and it bears 70% of the body’s immune defense function. Any disruption of barrier integrity leads to metabolic dysfunction of the organism and affects intestinal health (Camilleri et al. 2012). However, SCFAs are essential as signaling molecules in the intestinal barrier.

### Modulation of the microbial barrier by SCFAs

The mammalian gastrointestinal tract is inhabited by hundreds of millions of microbiota, which play critical regulatory roles in physiological processes such as nutrient digestion, intestinal barriers, immune responses, and endocrinology. However, dysregulation of gut microbial transport can impair the intestinal physical barrier and immune dysfunction, impairing animal intestinal health (Beaumont et al. 2020). SCFAs, as metabolites of microbes, play an essential role in intestinal biobarriers. SCFAs undergo β-oxidation in colonocytes that promote hypoxia in the lumen of the colon and prevent the development of parthenogenetic anaerobic bacteria (e.g., pathogenic *Escherichia coli* and *Salmonella*) expansion while protecting the host from the expansion of potentially pathogenic bacteria by increasing intestinal oxygen utilization leading to a decrease in the availability of oxygen in the gut (Byndloss et al. 2017; Topping and Clifton 2001). Liu et al. (2023) showed that the addition of 0.2% sodium butyrate in a weaned piglet model increased the abundance of microbes and probiotic diversity in the gut, promoting *Lactobacillus*, *Megasphaera*, and *Blautia*. In the LPS-induced piglet model, the addition of butyrate resulted in a higher relative abundance of beneficial bacteria *Firmicutes*, *Bacteroidetes*, *Clostridiaceae*, *Lactobacillus*, and *Prevotella* but a lower abundance of harmful bacteria Proteobacteria, Enterobacteriaceae, and *Escherichia–Shigella* (Han et al. 2022). In addition, microbes in the intestinal tract, such as *Bifidobacteria*, *Lactobacillus*, *Firmicutes*, and *Bacteroidetes*, act as fiber-degrading bacteria that promote DF fermentation to produce SCFAs (Bach Knudsen et al. 2018; Markowiak-Kopeć and Śliżewska 2020). In conclusion, SCFAs and intestinal microbes interact, thus achieving intestinal ecological balance.

### Modulation of chemical barriers by SCFAs

The intestinal chemical barrier consists mainly of a mucus layer containing mucin (MUC) secreted by specialized cup cells of the intestinal mucosa (Pelaseyed et al. 2014). The intestinal mucus layer is the first line of defense that protects the intestinal epithelium from pathogenic microbes and effectively prevents the entry of chemicals, toxins, pathogens, and allergens into the organism (Di Tommaso et al. 2021). MUC secreted by goblet cells effectively enhances the mucus barrier on the epithelial surface. It protects the epithelial cells from potential pathogens and other deleterious factors in the lumen, thus creating a safe microenvironment for colonic epithelial cell differentiation (Khan et al. 2022). SCFAs activate inflammatory vesicles in intestinal epithelial cells, promote the production of anti-inflammatory factors, and upregulate the expression of MUC genes in the intestinal tract, thereby enhancing the intestinal chemical barrier function (Ma et al. 2022b; Sun et al. 2017). One to fifteen millimolar propionate and 1 nM butyrate can stimulate MUC2 gene expression through MUC2 gene histone acetylation/methylation (Burger-van Paassen et al. 2009). Diao et al. (2019) reported that gavage of SCFAs in weaned piglets enhanced the intestinal chemical barrier function by stimulating the expression of intestinal MUC1 and MUC2 genes through the mitogen-activated protein kinase (MAPK) signaling pathway. In addition, acetate and propionate can exert antimicrobial effects by promoting the release of host antimicrobial peptides (Fukuda et al. 2011). Butyrate triggers the induction of a-defensin or antimicrobial peptide secretion by Paneth cells in the small intestine (Takakuwa et al. 2019). Therefore, SCFAs can enhance the chemical barrier of the intestine through multiple pathways, mainly by promoting the expression of MUC-related genes, which in turn enhances the chemical barrier of the animal intestine.

### Modulation of mechanical barriers by SCFAs

Intestinal epithelial cells and the tight junctions between them are essential components of the mechanical barrier, and evidence suggests that enhanced expression of tight junction proteins (TJs) plays a crucial role in maintaining the mechanical barrier in the animal intestine and inhibiting pathogen invasion into the organism (Ma et al. 2022b). TJs, as a significant determinant of the physical barrier of the intestine, consist of multiple-protein complexes with different functions located in the apical portion of the lateral membrane of intestinal epithelial cells, which is mainly composed of transmembrane proteins, such as Claudin, Occludin, and Zonula Occludin (ZO), play essential roles in maintaining the intestinal mechanical barrier and regulating intestinal permeability (Vancamelbeke et al. 2017). Butyrate, one of the most studied of the many SCFAs promotes the expression of TJ proteins and maintains intestinal homeostasis by inhibiting HDAC (Gao et al. 2021). Similarly, Huang et al. (2015) found that sodium butyrate significantly increased the expression of jejunal and colonic Occludin proteins in weaned piglets and reduced diarrhea by decreasing intestinal permeability. In addition, Tong et al. (2016) found that propionate increased the expression of intestinal ZO-1 and Occludin, ultimately improving intestinal health. SCFAs also activated the AMPK pathway to upregulate the expression of ZO-1 in intestinal epithelial cells and protect the integrity of the intestinal barrier (Voltolini et al. 2012). In conclusion, SCFAs regulate the mechanical barrier function of the animal intestine by promoting the expression of TJs and reducing intestinal permeability.

### Modulation of the immune barrier by SCFAs

The intestinal immune barrier is a well-developed and complex local immune system, including intestinal mucosa-associated lymphoid tissues and immune cells, which play a role in the removal of antigens by secreting a variety of cytokines and immunoglobulins that block the adhesion of various pathogenic microbial antigens to the intestinal mucosa (Gou et al. 2022). SCFAs produced by intestinal microbial metabolism are involved in regulating the functions of immune and non-immune cells in the intestinal mucosa through two pathways, namely, activation of different GPRs to stimulate cellular signal transduction and inhibition of HDACs to regulate gene expression, affecting immune cell gene expression, chemotaxis, differentiation, proliferation, and apoptosis, and thus regulating the intestinal immune barrier function (Sadler et al. 2020).

GPRs are widely distributed in a wide range of immune cells and intestinal epithelial cells. They regulate almost all cellular and physiological functions in the organism. However, different SCFAs can bind and activate different types of receptors and thus function, among which acetate, propionate, and butyrate can activate GPR41/GPR43, and GPR109A can only be activated by butyrate (Xu et al. 2020b). In the early stage of inflammation, SCFAs activate GPR41, and GPR43 on the surface of intestinal epithelial cells activates MAPK signaling and promoting the rapid production of chemokines and cytokines (Kim et al. 2013). For example, acetate and propionate inhibit the expression of inflammatory factors, interleukin-6 (IL-6), IL-8, IL-1β, and tumor necrosis factor α (TNF-α) by activating GPR43 on the surface of intestinal epithelial cells acting on macrophages, thereby alleviating intestinal inflammation (Mizuta et al. 2019; Pirozzi et al. 2018). In addition, SCFAs activate GPR109A on the surface of dendritic cells (DCs) to induce the differentiation of regulatory T-cells and IL-10-secreting Treg cells and inhibit the expression of inflammatory cell Th17, thus collectively suppressing the progression of inflammation (Myunghoo et al. 2016; Park et al. 2015).

SCFAs act as ligands for HDAC inhibitors, and by inhibiting the action of HDACs on monocytes and neutrophils, they lead to the inactivation of NF-κB, reduce the expression of inflammatory factors, and enhance the immune response, which helps the body to rapidly clear pathogens and shorten the time of inflammatory response (Meng et al. 2018; Rooks and Garrett 2016). In addition, T-cell differentiation can be accomplished by the action of butyrate-mediated HDACs, which prevent the degradation of Foxp3 by affecting its acetylation, and butyrate, by inhibiting HDACs, prevents proteasomal degradation and enhances the stability and activity of Foxp3, which in turn increases the expression of the Foxp3 gene in Treg cells, thereby stopping the inflammatory response (Smith et al. 2013). SCFAs also directly activate the mammalian target of the rapamycin pathway in intestinal B cells, increase glucose uptake and glycolytic activity, and promote B cell differentiation into sIgA-producing plasma cells (Nastasi et al. 2015). In addition, SCFAs promote the cytosolic transport of intestinal epithelial cells, increase the content of sIgA in the intestinal mucosa, agglutinate bacteria and adhere them to the mucus, preventing direct contact between bacteria and the surface of intestinal epithelial cells, and prevent bacterial invasion and infection (Yao et al. 2022).

In addition to promoting gut health as signaling molecules, SCFAs are also involved in energy metabolism and appetite regulation by improving the body’s glucose homeostasis and insulin sensitivity. SCFAs trigger host signaling through the activation of GPRs or inhibition of HDAC, which in turn stimulates the peptide YY (PYY) and glucagon-like peptide1 (GLP-1), two enteric hormones that act on the hypothalamus, which can transmit satiety messages and increase the sense of satiety in the body (Akhlaghi M. 2022). In a porcine model, cecum injection of propionate stimulates the secretion of satiety hormones by colonic tissues and regulates the expression levels of neuropeptides involved in appetite control via the brain-gut axis to reduce short-term feed intake (Zhang et al. 2022). Jiao et al. (2020) showed that dietary SCFAs can increase serum GLP-1, PYY, leptin, and other hormones to regulate appetite and control weight. Secondly, SCFAs regulate body lipid metabolism and muscle fiber formation through different pathways, thus improving meat quality. Jiao et al. (2021) showed that injecting SCFAs in the ileum can improve carcass traits and meat quality by regulating lipid metabolism. In conclusion, the effects of different SCFAs and combinations on lipid metabolism, meat quality traits, and their regulatory effects on appetite must be further explored.

**Fig. 2:**
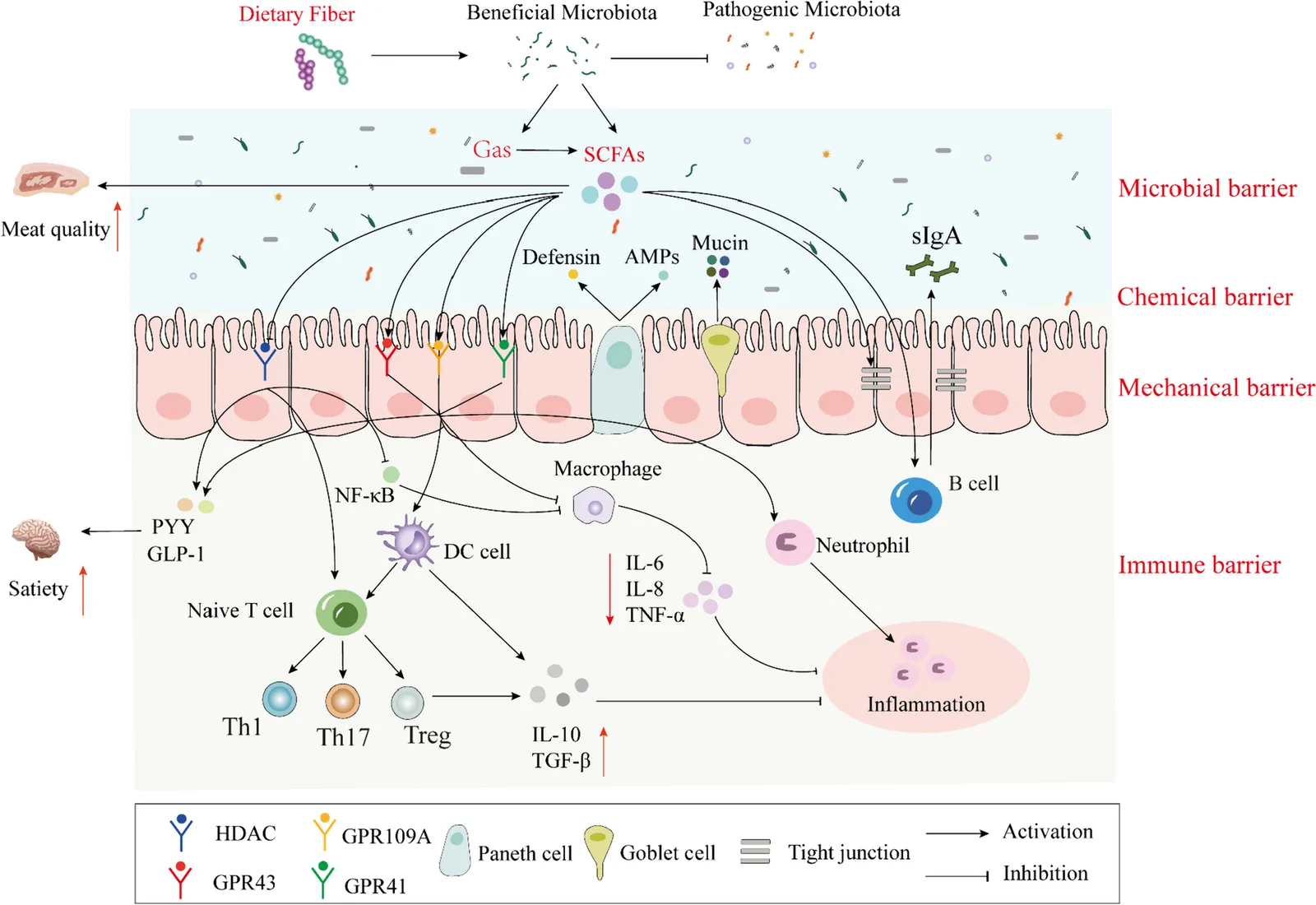
Immune regulation mechanism of dietary fiber and its metabolites on the intestinal tract

Gases and SCFAs, as the primary metabolites of intestinal microbial fermentation DF, are crucial in alleviating intestinal inflammation, maintaining intestinal barrier function, and improving meat quality (Fig. 2). As a new type of green feed additive, they have broad application prospects in livestock and poultry production. However, there are still many problems to be solved in practical applications. For example, optimizing its addition method in livestock and poultry diets requires further research to determine the appropriate addition level. In addition, the mechanism of regulating host immune function and inhibiting the occurrence of intestinal diseases still needs further study. An in-depth understanding of these mechanisms will help to make more effective use of them and provide new ideas for the prevention and treatment of diseases.

## The role of DF in pig nutrition

In the past, DF was generally considered a complex that is not easily degraded by endogenous digestive enzymes in monogastric animals and, therefore, can reduce the digestibility of feed (Li et al. 2021). With a deepening understanding of the function of DF and the potential health benefits of animals, people began to pay more attention to the application of fermentable DF components in pig diets. Through continuous improvement in feed formulation and intensive research on dietary fiber, the addition of moderate amounts of DF to monogastric animal diets not only helps to reduce the use of conventional feeds and costs but also improves the gut health of the animals and enhances farming efficiency (Jha et al. 2019). This helps to improve the adverse effects of intensive farming models, such as improving the reproductive performance of sows, alleviating weaning stress syndrome in piglets, and improving the meat quality of fattening pigs.

## Effects of DF on intestinal health and reproductive performance of sows

Under the current production conditions, restricted feeding and single-column feeding are usually selected to maintain the standard fat shape and reproductive performance of pregnant sows. However, over-restricted feeding can cause sows to be hungry and show abnormal behaviors such as chewing the vacuum, getting up, and lying down frequently (Tian et al. 2020). With the rapid development of the fetus, the metabolism and immunity of sows in late pregnancy will undergo dramatic changes, which will lead to oxidative stress, inflammatory response, and intestinal microbes disorder, which in turn will lead to reduced feed intake of sows, blocked fetal development, and even abortion (Li et al. 2022b). In addition, the amount of feed intake during pregnancy is closely related to free feeding during lactation. Malnutrition during lactation can destroy the normal development of offspring and affect their growth and development (Cheng et al. 2018). Therefore, alleviating the adverse reactions caused by starvation during pregnancy and ensuring the body’s regular metabolism and immune response is essential for the growth and development of sows and their offspring.

The addition of DF has been shown to produce satiety, reduce the harm of feed restriction, improve animal welfare, and enhance reproductive capacity (Table 2). DF can increase chewing activity and saliva production through its expansion ability, delay gastric emptying speed, and stimulate the central nervous system to produce satiety (Huang et al. 2020; Tian et al. 2020). In addition, the produced SCFAs trigger the host signal by activating GPRs or inhibiting HDAC, thereby regulating the release of intestinal hormones GLP-1 and PYY so that the body has a sense of satiety (Byrne et al. 2015). DF can also improve metabolic syndrome in late pregnancy through microbial remodeling, reduce inflammation and oxidative stress, and improve the body's immunity (He et al. 2015). The addition of DF can improve the composition and diversity of microbes, and increase the beneficial microbes (*Lactobacillus*, *Ruminococacae*, etc.) in the intestine, and reduce the proportion of harmful bacteria (*Desulfovibrio*, etc.) (Gao et al. 2023; Lu et al. 2022; Shang et al. 2021b; Xu et al. 2020a). The gut microbiota of sows is also transmitted to offspring through the entero-mammary axis (Rodríguez 2014), forming the early gut microbiota of offspring, which drives the early innate immune development and metabolic phenotype after birth (García-Mantrana et al. 2020; Wu et al. 2020). The addition of SDF (2.0% pregelatinized waxy maize starch plus guar gum) in late pregnancy improved the disease resistance of piglets by changing the intestinal microflora (*Lactobacillus* and *Bacteroides* increased significantly) (Cheng et al. 2018). Similarly, Liu et al. (2021) showed that adding IDF (alfalfa meal) to sows during pregnancy gave similar results. Moreover, supplementation of DF in late pregnancy significantly improved the antioxidant capacity of sows (Liu et al. 2020). This antioxidant capacity can also be transferred to piglets through milk to enhance their antioxidant capacity (Chen et al. 2019b; Chen et al. 2020a). Li et al. (2020) confirmed this conclusion by adding DF (inulin and cellulose) to the diet of pregnant sows. At the same time, adding DF can also improve the survival rate of embryos during pregnancy, reduce the proportion of stillbirths and the total mortality rate of suckling piglets, and increase the birth rate of piglets (Feyera et al. 2017). Oocytes are very sensitive to maternal nutrient levels. The intake of DF can change the levels of physiological hormones and metabolites, thus affecting the function of the ovary (Ashworth et al. 2009). Men et al. (2022) found that adding DF (inulin and cellulose) to the diet can promote the maturation of oocytes, increase the survival rate of embryos, and increase the litter size of sows. DF can also effectively improve the quality of colostrum and regular milk and the lactation yield of lactating sows (Feyera et al. 2019; Loisel et al. 2013). DF can synthesize the precursor of milk fat through its metabolite SCFAs and provide sufficient nutrition to piglets through the colostrum to improve the growth performance of piglets (Chen et al. 2019a; Chen et al. 2019b; Chen et al. 2020a). In conclusion, DF is closely related to the gut microbiota, antioxidant capacity, inflammatory response, and sow production performance.

**Table 2:** Effects of dietary fiber on reproductive performance and gut microbiota and metabolites in sows

Feeding phase	Fiber sources and level	Mechanism		Main results	References
		SCFAs	Gut microbiota		
Sow (70 day of gestation)	5% resistant starch die	N/A	N/A	Satiety↑; stillbirth number↓; stillbirth rate↓; serum peptide YY↑; glucagon-like peptide-1↑	(Huang et al. 2020)
Sow (112 day of gestation)	2% resistant starch	Propionate↑ Butyrate↑	*Lactobacillaceae*↑ *Desulfovibrio*↓	IL-10↑, TNF-αin serum↓; stillbirth number↓	(Lu et al. 2022)
	2% konjaku flour	Propionate↑ Butyrate↑	*Lactobacillus*↑ *Desulfovibrio*↓	IL-10↑, TNF-α in serum↓; stillbirth number↓	(Lu et al. 2022)
Sow (110 day of gestation)	20% sugar beet pulp	Acetate↑ Butyrate↑	*Christensenellacae_R-7_group*↑ *Ruminococacae_UCG002*↑ *Terrisporobacter*↓	Lactation birth weight loss↓; total cholesterol and non-esterified fatty acids↓; IL-6 and TNF-α in serum↓;	(Shang et al. 2021b)
Sow (109 day of gestation)	2% guar gum plus pregelatinized waxy maize starch (SF)	Propionate↑	*Ruminococacae*↑ *Clostridium*↓	Insulin sensitivity↑; systemic inflammation↓	(Xu et al. 2020a)
Sow (56 day of gestation)	Soybean hulls high-fiber (8% crude fiber)	N/A	*Proteobacteria*↓ *Lactobacillaceae*↑ *Lactobacillus*↑	Litter weight and average daily feed of piglets↑; immunoglobulins in colostrum↑	(Gao et al. 2023)
Sow (110 day of gestation)	3% (50% guar gum + 50% cellulose)	Propionate↑ Butyrate↑	*Bacteroides*↑ *Eubacterium-hallii-group*↑ *Roseburia*↑	Sow diarrhea rate↓ Stillbirth number↓	(Wu et al. 2020)
Sow (100 day of gestation)	5% alfalfa meal	Butyrate↑	*Prevotellaceae_NK3B31_group*↑ *Terrisporobacter*↓	ROS, endotoxin, IL-6, and TNF-α in serum↓, IL-10 in serum↑	(Liu et al. 2021)

*N/A* not available

In summary, adding DF to the diet of pregnant sows can promote intestinal health and improve the reproductive performance of sows. However, the sources and components of DF are relatively wealthy and complex, and more data is needed to form a reference standard to determine DF’s addition level and optimal addition time. In addition, the research on the effects of DF types on the gut microbiota of sows is still in the preliminary exploration stage, and a large amount of data is still needed to enrich the content of this aspect. Therefore, in the future, it is necessary to study further and explore the effects of dietary fiber on pregnant sows and their offspring to formulate a more scientific and reasonable feeding plan.

## Effects of dietary fiber on the weaning stress of piglets

Early weaning in modern intensive breeding is widely used to shorten sows’ breeding cycle, improve sows’ production capacity, feed and breeding equipment utilization, and benefit breeding enterprises more economically (Tang et al. 2022). However, due to immature intestinal development and incomplete digestive and immune systems in piglets, sudden maternal separation, and changes in the environment and feed morphology can lead to a strong stress response (Su et al. 2022). This stress response may lead to intestinal microbes imbalance, intestinal morphological damage, and barrier dysfunction, which in turn leads to weaning stress syndrome characterized by decreased nutrient absorption rate, increased diarrhea rate, growth retardation, and severe piglet death (Tang et al. 2022). Therefore, it is essential to correctly understand the effect of weaning stress on intestinal health and improve the intestinal barrier damage caused by weaning stress through reasonable nutritional regulation to improve the production efficiency of animal breeding.

The intestinal tract is the central part of digestion and absorption of nutrients in piglets, and it is also the body’s largest immune organ. As the first line of defense against the invasion of pathogens in vitro, its structural and functional integrity is the premise for the regular and healthy growth of weaned piglets (Wang et al. 2016). When weaned piglets are subjected to weaning stress, the intestinal environment is vulnerable to the invasion of pathogenic microbes such as *Escherichia coli* and *Actinobacillus*, which stimulate the secretion of inflammatory factors in the intestinal mucosa, destroy the intestinal morphology, and damage the intestinal mucosal barrier function (Gresse et al. 2017; Karasova et al. 2021; Su et al. 2022; Tang et al. 2021). However, adding DF to the diet has become an effective strategy to alleviate weaning stress (Table 3). Studies have shown that the addition of DF (inulin, wheat bran, and alfalfa meal, etc.) to the diet can improve the microbial structure and increase the abundance of beneficial bacteria and the content of metabolites, and improve the intestinal health of piglets (Chen et al. 2020b; Dang et al. 2022; Liu et al. 2018; Molist et al. 2010). Wang et al. (2020) reported that the addition of inulin to the diet did not affect the growth performance of piglets. It improves intestinal morphology and microbial composition and improves intestinal health by increasing the number of cecal *Lactobacillus* and decreasing *Escherichia coli* to enhance the production of SCFAs. Chen et al. (2013) also showed that the addition of wheat bran (mainly IDF) improved intestinal morphology and intestinal microbes, increasing the abundance of beneficial bacteria (*Lactobacillus* and *Bifidobacterium*), which in turn came to alleviate intestinal inflammation and improve the integrity of the intestinal mucosal barrier. It can be seen that DF promotes growth and development by improving intestinal microbes and increasing the production of metabolites, improving the morphology of intestinal mucosal epithelium, restoring intestinal barrier function, and improving the ability to digest and absorb nutrients.

**Table 3:** Effect of dietary fiber on growth performance and intestinal microbes and metabolites in piglets

Growth phase	DF	Mechanism		Main results	References
		SCFAs	Gut microbiota		
Weaned piglets (21 d)	4% pectin	Acetate↑ Propionate↑ Butyrate↑	*Lactobacillus↑* *Clostridium_sensu_stricto_1↑* *Blautia↑* *Streptococcus↓*	Goblet numbers↑; proinflammatory factor (IL-1β, IL-6, IL-18, and TNF-α) in the serum↓; tight junction protein (ZO-1, Muc2)↑	(Dang et al. 2022)
Weaned piglets (32 d)	5% alfalfa meal	Propionate↑	*Lactococcus↑* *Enterococcus↑*	Diarrhea rate↓; piglet mortality↓	(Liu et al. 2018)
Weaned piglets (21 d)	0.25% inulin	Acetate↑ Propionate↑	*Lactobacillus↑* *Escherichia coli↓*	No effect on the growth performance; VH and VCR↑; insulin-like growth factor -1↑, TNF-α in the intestinal mucosa↓	(Wang et al. 2020)
Weaned piglets (28 ± 2 d)	10% wheat bran	N/A	*Lactobacillus↑* *Bifidobacterium↑* *Escherichia coli↓*	VCR↑; TJ distribution and abundance↑; piglets diarrhea rate↓	(Chen et al. 2013)
Weaned piglets (28 d)	4% sugar beet pulp	N/A	*Lactobacillus↑*	N/A	(Shang et al. 2021a)
	6% wheat bran	Butyrate↑	*Lachnospira↑* *unclassified_f_Lachnospiracea↑*	Average daily gain and gain: feed↑; piglets diarrhea rate↓	(Shang et al. 2021a)
Weaned piglets (28 d)	6% sugar beet pulp	Acetate↑	*Lactobacillus↑*	Nutrient digestibility (dry matter, organic matter, gross energy, neutral detergent fiber, acid detergent fiber)↓	(Shang et al. 2020)

*N/A* not available

However, we found some differences between different types of DF in regulating the intestinal barrier, nutrient metabolism, and growth and development of piglets. SDF is susceptible to fermentation by intestinal microbes in the hindgut and alleviates diarrhea, mainly through its metabolites and by reducing the proliferation of pathogenic microbes; however, it also increases the viscosity of digestive juices and reduces nutrient utilization (Dong et al. 2019). In contrast, after reaching the gastrointestinal tract, IDF can stimulate the peristalsis of the digestive tract, reduce the proliferation of pathogens in the gastrointestinal tract, and have a water-binding capacity, reducing diarrhea incidence (Canibe et al. 2022). At the same time, IDF has an anti-nutritional effect and is slowly fermented in the hindgut of the animal, supplying less energy for the organism (Champ et al. 2003). Shang et al. (2021a) investigated the effects of 6% wheat bran (IDF-based) helped to increase the daily feed intake and the feed-to-weight ratio of piglets and reduced piglet diarrhea by modulating the gut microbial structure and intestinal morphology. On the contrary, 4% of sugar beet pulp (SBP, SDF-based) showed a decrease in daily weight gain and feed-to-weight ratio. Similarly, it was demonstrated in another paper that the addition of 6% SBP reduced nutrient digestibility yet significantly increased the abundance of the beneficial bacterium *Lactobacillus* (Shang et al. 2020). These may be related to the SDF properties in SBP (Flis et al. 2017). Chen et al. (2020b) reported that the addition of inulin (SDF-based) and lignocellulose (IDF-based) with consistent total fiber to piglet diets revealed that inulin was effective in improving the microbial composition of the hindgut of piglets, promoting hindgut fermentation and enhancing the colonic barrier function. However, IDF-based lignocellulose was more effective than inulin in improving growth performance and promoting nutrient digestion and absorption. It can be seen that the addition of moderate levels of IDF or slow-fermenting DF to the diet 2 weeks before to weaning modulates the physicochemical specialties of the chow enhances the fermentation capacity of the pig and competes for adherence sites on the mucosa of the gastrointestinal tract for pathogenic bacteria.

In conclusion, different DFs alleviate intestinal inflammation and barrier damage induced by weaning stress through their unique mechanisms of action, thereby maintaining intestinal health. However, a comprehensive evaluation of specific fiber material properties, diet formulation structure, and feeding stage is the key to accurately selecting suitable fiber materials for piglets. Therefore, these factors must be considered when formulating feeds to ensure piglets’ gut health and growth.

## Effect of dietary fiber on growth performance and meat quality in fattening pigs

With the rapid development of breeding technology, the modern mode of pursuing efficient growth and a high lean meat rate profoundly affect the quality and nutritional value of pork (Yu et al. 2020a). In this process, although animal genes determine their growth status, the importance of nutritional regulation in animal production must be addressed, and its impact on pork quality is crucial (Rauw et al. 2020). Secondly, the hindgut of pigs is well developed, the intestinal flora is stable, and the utilization rate of feed is high. Therefore, as an essential part of feed, fiber has become an important research topic in the breeding industry to study its effect on the growth performance and meat quality of fattening pigs and to improve the quality and nutritional value of pork through reasonable feed formulation and nutritional regulation to meet the needs of consumers.

Studies on the effect of DF on pig growth performance were previously reviewed by Agyekum (Agyekum and Nyachoti 2017), which showed that most of the feed ingredients, regardless of whether they are enriched with SDF or IDF, do not promote or even inhibit growth performance, mainly since high-fiber diets inhibit the deposition of lean meat in growing pigs (De Jong et al. 2014; Magistrelli et al. 2009), but that the addition of DF improves microbial structure, barrier function, and promotes intestinal health (Laitat et al. 2015; Zhao et al. 2019). On this basis, we further reviewed the effects of different fibers on growing pigs’ growth performance and meat quality, focusing on the latter, as shown in Table 4. Diao et al. (2020) added 5.74% SBP to fattening pigs, which reduced weight gain and daily feed intake. However, the expression of genes related to intestinal weight and intestinal barrier function increased significantly. Ma et al. (2022a) showed that adding mulberry leaf powder fiber had no effect on the growth performance but improved their intestinal health by increasing the beneficial microbial *Bifidobacteria*. He et al. (2018) added that 10% oat bran did not affect growth performance. It also increases the fiber-degrading bacteria (*Prevotella*, *Butyricicoccus*, and *Catenibacterium*) producing SCFAs and improving gut health. Wang et al. (2019b) showed that the addition of 0.5% inulin to the diet significantly increased average daily weight gain, serum growth hormone, and insulin. In summary, DF did not affect the growth performance of growing pigs or even reduced feed utilization, mainly due to the reduction of nutrient digestibility and energy deposition induced by DF; however, a small number of studies have shown that DF can help improve growth performance, possibly by improving intestinal health.

**Table 4:** Effect of dietary fiber on growth performance and intestinal microbes and metabolites in growing pigs

Growth phase	DF	Mechanism		Main results	References
		SCFAs	Gut microbiota		
Growing pigs (BW = 45.8 ± 2.78 kg)	5.74% sugar beet pulp	Acetate↑ Butyrate↑	*Lactobacillus*↑ *Bifidobacterium*↑ *Enterobacteriaceae*↓ *Escherichia coli*↓	Average daily gain, average daily feed intake, feed: gain, feed digestibility, and growth performance↓; Intestinal barrier and intestinal health↑	(Diao et al. 2020)
Growing pigs (BW = 72.2 ± 4.8 kg)	9% mulberry leaf	N/A	*Bifidobacterium*↑ *Campylobacter*↓	No negative effects on growth performance; serum immunoglobulin M↑; a* value ↑; Occludin and Muc-2↑	(Ma et al. 2022a)
Growing pigs (BW = 30.5 ± 2.6 kg)	10% oat bran	Propionate↑	*Prevotella*↑ *Butyricicoccus*↑ *Desulfovibrio*↓	Not affect dietary nutrient digestibility and growth performance; IL-8, NF-κB, and TNF-α gene levels in the colon↓	(He et al. 2018)
Growing pigs (BW = 22.0 ± 1.0 kg)	0.5% inulin	N/A	N/A	Average daily gain, dressing percentage, the loin-eye are and growth performance↑	(Wang et al. 2019b)
Growing pigs (BW = 40.5 ± 0.63 kg)	15% mulberry leaf	N/A	N/A	Growth performance, average daily gain, carcass weight, and dressing percentage↓; Pork quality↑, lower backfat depth, shear force, and drip loss↓; MyHCI, MyHCIIa and intramuscular fat↑	(Zeng et al. 2019)
Growing pigs (BW = 93.30 ± 1.60 kg)	4% mulberry leaf	N/A	N/A	a* value↑; shear force and drip loss↓; MyHCI and MyHCIIa mRNA levels↑	(Chen et al. 2021)
Growing pigs (BW = 77.79 ± 6.97 kg)	30% alfalfa meal	N/A	*Prevotella-2*↑ *Faecalicatena*↑	Not affect growth performance; a*value, b*value and essential amino acid↑	(Li et al. 2022a)
Growing pigs (BW = 25 kg)	100 mg/kg β-glucan	N/A	N/A	Drip loss↓; muscle pH and a* value↑; intramuscular fat↑; saturated fatty acid and polyunsaturated fatty acid↑	(Luo et al. 2019)
Growing pigs (BW = 40 kg)	7% bran fiber	N/A	N/A	Not affect carcass weight; MyHCIIb and MyHCIIx mRNAs and protein levels↓; MyHCI mRNA levels↑	(Han et al. 2020)

*N/A* not available, *BW* birth weight

As an essential indicator of pig production, pork quality is determined by two main aspects on the one hand, the physicochemical properties of pork, such as shear force, drip loss, meat color, and pH value. Secondly, it is evaluated by the nutrients present in the pork, such as fatty acids and amino acids (Grela et al. 2009). Zeng et al. (2019) and Chen et al. (2021) showed that the addition of mulberry leaf fiber of fattening pig increased meat color, reduced cooking losses and drip losses, and improved the quality of the meat by altering myofiber profiles. In addition to physical and chemical indicators, the nutritional components in pork, such as intramuscular fat (IMF) content, are also important indicators for evaluating meat quality. Fatty acid is an important chemical substance that constitutes fat. The regulation of IMF on meat quality is the role of fatty acids. In addition, the content and composition of free amino acids in muscle, especially flavor amino acids, play a decisive role in meat’s nutritional value and directly affect pork’s flavor characteristics (Ma et al. 2020). Li et al. (2022a) showed that the addition of 30% alfalfa meal to black-covered pigs (Chinese local pigs) increased the a*, b* value of longissimus dorsi muscle, as well as flavor amino acids and essential amino acids. Luo et al. (2019) found that 100 mg/kg β-glucan dietary supplementation significantly increased IMF content and improved the proportion of saturated fatty acid and polyunsaturated fatty acid in finishing pigs. Furthermore, myofibrils constitute the main component of muscle and play an essential role in regulating meat quality (e.g., pH and meat color) (Han et al. 2020). They are classified into slow-contracting oxidative fibers (MyHCI), fast-contracting oxidative fibers (MyHCIIa), fast-contracting glycolytic fibers (MyHCIIb), and fast-contracting oxidative glycolytic fibers (MyHCIIx) based on their morphological characteristics and oxidative and glycolytic metabolism capacity, which are related to meat quality traits (Hu et al. 2008). It was shown that the addition of 7% bran fiber to the feed reduced the expression of MyHCIIb and MyHCIIx in the Erhualian pig (a Chinese domestic pig), accompanied by an increase in the mRNA expression of MyHCI in the longest muscle of the back trend (Han et al. 2020). Li et al. (2015) showed that low-starch and high-fiber diets significantly increased the expression of oxidized myofibrils (MyHCIIa and MyHCI) and decreased the expression of glycolytic myofibrils (MyHCIIb and MyHCIIx) in longissimus dorsi muscle. Compared to MyHCIIb-type fibers, MyHCI-type fibers have a finer diameter, lower shear, and better tenderness, and MyHCI is rich in myoglobin and mitochondrial oxidative metabolic enzymes, as well as lower glycogen and lactic acid levels, which are also closely related to meat quality (Hanna et al. 2023). At the same time, oxidized muscle fibers have relatively higher lipid and phospholipid content than glycolytic muscle fibers (Guo et al. 2020). Thus, DF improves meat quality by promoting the content of oxidized muscle fibers (MyHCI and MyHCIIa). In addition, SCFAs can improve meat quality by regulating lipid metabolism, but the specific mechanism remains to be further studied (Jiao et al. 2021).

In conclusion, although DF has a low impact on growth performance and even reduces feed utilization in fattening pigs, it significantly improves meat quality. This is closely related to the production of SCFAs by DF fermentation in the hindgut, which can regulate the energy metabolism of muscle cells by affecting the synthesis and function of mitochondria, and this is also a potential mechanism by which DF interferes with muscle fiber composition and fat content. However, there are relatively few studies on the effects of DF on meat quality, and further research is needed.

With continuous and in-depth research on the physicochemical properties of DF and animal nutrition, we have gained a more comprehensive understanding of DF’s nutritional and anti-nutritional functions in the porcine intestinal tract. More and more fiber sources, such as wheat bran, inulin, sugar beet meal, and alfalfa grass meal, have been extensively studied in pigs. For sows, the addition of DF helps to improve sow intestinal health and reproductive performance, as well as piglet intestinal health and growth performance through mother-to-child transfer. The potential value of DF for weaned piglets has also been studied extensively, mainly including the improvement of gut health and reduction of diarrhea in weaned piglets. In addition, the pros and cons of different types of DE (IDF and SDF) were further considered, and the addition of IDF was found to be more suitable for the growth of weaned piglets. DF has a relatively small effect on growth performance for growing pigs but can positively affect meat quality by improving fatty acid, amino acid, muscle fiber composition, etc. However, the specific mechanism still needs further research.

## Conclusion and future

**Fig. 3:**
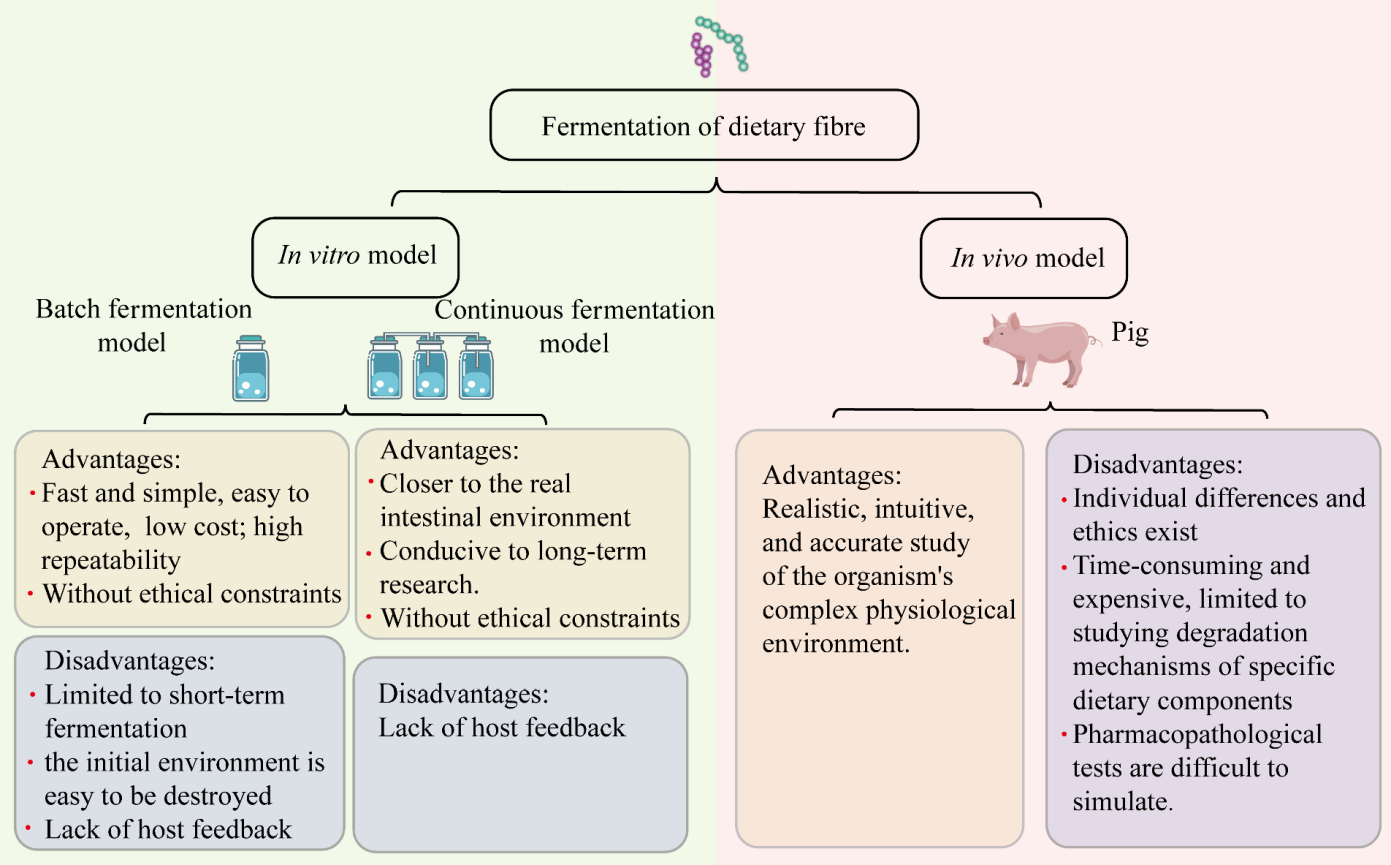
Advantages and disadvantages of in vivo and in vitro fermentation models (Comino et al. 2018; Maccaferri et al. 2010; Wang et al. 2019a)

In conclusion, we summarized the fermentation properties of DF and the metabolic mechanisms of its metabolites by in vitro models, demonstrating the critical role of SCFAs and gases in improving the organism’s health. At the same time, some information about host-microbe interactions can be improved through traditional animals in vivo models, and the most direct feedback on nutrients is generated, confirming the beneficial effects of DF on sow reproductive performance, piglet gut health, and growing pork quality. Subsequently, Fig. 3 compares the advantages and disadvantages of different models to deepen the understanding of DF fermentation by different models. However, both in vivo and in vitro models have advantages and disadvantages. Therefore, the ultimate method for studying the future relationship between fiber-animal gut microbiota metabolites should combine in vitro and in vivo models to produce complementary effects. For example, the correlation between fiber and animal gut microbiota can be first established by animal studies, and the subsequent mechanism can be verified by in vitro models. They combine in vivo experiments with in vitro models to gain an in-depth understanding of the physiological characteristics of dietary fibers and achieve “precise nutrition.”

## Data Availability

Not applicable.
